# Analysis of risk factors associated with secondary open-angle glaucoma in Posner-Schlossman syndrome: A retrospective case-control study

**DOI:** 10.3389/fmed.2022.1064449

**Published:** 2023-01-09

**Authors:** Jiajun Li, Yuke Ji, Weihua Yang, Yujia Yao, Suyu Wang, Ziran Zhang, Jin Yao, Keran Li

**Affiliations:** ^1^The Fourth School of Clinical Medicine, Nanjing Medical University, Nanjing, China; ^2^Department of Ophthalmology, The Affiliated Eye Hospital of Nanjing Medical University, Nanjing, China

**Keywords:** Posner-Schlossman syndrome, open-angle glaucoma, anterior uveitis, intraocular pressure, interleukin, cytomegalovirus (CMV)

## Abstract

**Background:**

Posner-Schlossman syndrome (PSS) is a relatively rare cause of chronic secondary open-angle glaucoma (OAG), but the exact cause is unknown. This study aimed to determine potential risk factors for OAG secondary to PSS and to provide a basis for early intervention in the development of PSS.

**Methods:**

This was a retrospective case-control study. Nine cases diagnosed with PSS and seven cases diagnosed with OAG secondary to PSS were selected and their aqueous humor assays at the first occurrence of PSS were collected. Clinical characteristics including age, sex, disease duration, eye laterality, baseline visual acuity, maximum IOP, corneal endothelial cell density, visual field, retinal nerve fiber layer thickness, cup-to-disk ratio, keratic precipitates, anterior chamber inflammation, and aqueous humor cytokine assay results were compared between the two groups.

**Results:**

The cytomegalovirus (CMV) positivity was 55.60% in patients with PSS and 100% in patients with OAG secondary to PSS. Corneal endothelial cell density was lower in patients with CMV-positive PSS (*p* = 0.0116). Concentrations of basic fibroblast growth factor (bFGF), interleukin (IL)-6, and vascular cell adhesion molecule (VCAM) in patients with PSS and IL-8, IL-6, and VCAM in patients with OAG secondary to PSS were higher than standard reference values; and IL-8 concentration was significantly higher in patients with OAG secondary to PSS (*p* = 0.0229). There were significant positive correlations between IL-8 and IL-6, IL-6 and VCAM (*p* = 0.0304, *p* = 0.0172) and a significant negative correlation between bFGF and vascular endothelial growth factor (VEGF) (*p* = 0.0497). Simultaneous increase of IL-8 and IL-6 concentration levels could be used as a cytokine indicator to predict secondary OAG in patients with PSS (*p* = 0.0095).

**Conclusion:**

Simultaneous increase of IL-8 and IL-6 concentrations may be an important cause of accelerated secondary OAG in patients with PSS, with IL-8 playing a more critical role. IL-8 and IL-6 may be more reliable cytokine markers for predicting secondary OAG in PSS, However, the high possibility of secondary OAG in patients with CMV-positive PSS should not be ignored. Regulation of IL-8 and IL-6 levels may be a new strategy of preventing OAG secondary to PSS.

## 1. Introduction

Posner-Schlossman syndrome (PSS) was first described by Posner et al. (1) as a specific form of anterior uveitis with glaucoma primarily found in young adult men aged 20–50 years (1, 2). The main clinical features of PSS comprise recurrent episodes of unilateral acute elevation of intraocular pressure (IOP) with non-granulomatous uveitis. During acute attacks, the IOP of patients with PSS usually exceeds 40 mmHg, but the iridocorneal angle remains open and glaucoma-related parameters remain within the normal limits, with only mild symptoms such as blurred vision and ocular pain, resolving spontaneously within 1-2 weeks. Previously, the prognosis of patients with PSS was considered to be good, but it is now believed that PSS may be a relatively rare cause of chronic secondary open-angle glaucoma (OAG) (3, 4) that can lead to increased fluctuations in intraocular pressure, widening and deepening of the optic disk, thinning of the retinal nerve fiber layer, depression and atrophy of the optic nerve papillae, progressive visual field loss, and eventually irreversible visual impairment or even complete blindness, which cause severe stress and financial burden to patients and their families. A retrospective study found that approximately 26% of patients with PSS develop OAG, and the risk of developing OAG is 2.8 times higher in patients with PSS over 10 years than in those with PSS less than 10 years (4). Therefore, in order to control the progression of PSS, prevent further exacerbation of optic nerve damage, and improve the prognosis of patients, it is necessary to delineate the risk factors associated with the development of OAG in patients with PSS and to provide timely prediction, diagnosis, and intervention.

Intraocular fluid is a general term that includes aqueous humor, vitreous, and subretinal fluid. Owing to the blood-ocular barrier, intraocular fluid can better reflect changes in the intraocular microenvironment than blood, and its measurable components include nucleic acids of pathogenic microorganisms, antibodies, and cytokines. Aqueous humor is the most commonly used intraocular fluid for testing (5–9). With the development of precision medicine techniques, intraocular fluid testing has been widely used in the treatment of infectious eye diseases such as acute retinal necrosis, ocular toxoplasmosis, various types of uveitis, and other infectious eye diseases, which provides an important basis for clinicians to make accurate judgments and provide targeted treatment (10–12). Although there have been studies on intraocular fluid testing in patients with PSS and OAG (13–15), most have been limited to the correlation between intraocular fluid composition and the disease itself, and there have been no studies evaluating risk factors associated with OAG secondary to PSS. Therefore, the clinical characteristics of patients with PSS and patients with OAG secondary to PSS were analyzed, the relevant intraocular fluid components were quantified, and the associated risk factors in patients with OAG secondary to PSS were assessed in this study, intending to help clinicians make early diagnoses and effective interventions in patients with OAG that may be secondary to PSS, as well as to further improve the understanding of PSS.

## 2. Materials and methods

### 2.1. Subjects

This was a retrospective case-control study. Nine cases (9 eyes) with PSS and seven cases (7 eyes) with OAG secondary to PSS diagnosed between January 2019 and June 2022 at the Eye Hospital of Nanjing Medical University were selected. The PSS group included 8 males and 1 female, and the mean ± SD age was 46.56 ± 14.37 years; the OAG secondary to PSS group included 6 males and 1 female, and the mean ± SD age was 41.86 ± 10.79 years. Patients enrolled in this study met the following criteria (1, 2): (1) active PSS with recurrent episodes of elevated IOP (> 21 mmHg); (2) mild anterior chamber reaction with fine to medium-sized keratic precipitates; (3) unilateral involvement; (4) absence of peripheral anterior and/or posterior synechiae; (5) open iridocorneal angle and normal in appearance; and (6) all patients confirmed PSS or OAG secondary to PSS after diagnosis by glaucoma and uveitis specialists and evaluation by ophthalmic ancillary examinations. The exclusion criteria: (1) presence of other ocular diseases; (2) PSS under treatment or with poor control after short-term medication before the first episode; (3) OAG secondary to other known causes such as corticoid, ocular blunt contusions, and vascular disease; (4) other risk factors that may cause OAG, such as ocular hypertension, high myopia, and thick corneal thickness; (5) known systemic, hereditary, or infectious inflammation; (6) previous intraocular or extraocular surgery without complications; and (7) immunodeficiencies such as acquired immunodeficiency syndrome (AIDS) or history of cancer. The study was approved by the Institutional Review Board of the Eye Hospital of Nanjing Medical University. In accordance with the ethical guidelines of the Declaration of Helsinki, all clinical data and samples were collected with the written informed consent of patients and their families, and all patients were informed consent for the experimental data to be used in this study.

### 2.2. Clinical data

We collected the disease duration of all patients included in this study and all patients underwent comprehensive ophthalmologic examination during treatment at the hospital in accordance with clinical requirements, including slit lamp microscopy, indirect dilated ophthalmoscopy, peak IOP, corneal endothelial cell (CEC) density, baseline visual acuity, visual field, and retinal nerve fiber layer (RNFL) thickness. In addition, clinical findings such as cup-to-disk ratio, keratic precipitation, and anterior uveitis (Tyndall effect) were collected.

### 2.3. Aqueous humor

Aqueous humor collection was performed in all patients with the first occurrence of PSS. Aqueous humor collection method: Under ocular surface anesthesia, a 30-G syringe was inserted 1 mm inside the superior temporal corneal limbus parallel to the iris surface under a slit lamp microscope. In total, 70–100 μl of aqueous humor was extracted and transferred into 1.5-ml tubes. Samples were stored in a −80°C freezer and sent to the Beijing Giantmed Medical Laboratory for further analysis.

### 2.4. Aqueous cytokine and virus analyses

The concentrations of vascular endothelial growth factor (VEGF); vascular cell adhesion molecule (VCAM); serum basic fibroblast growth factor (bFGF); and the cytokines interleukin (IL)-6, IL-8, and IL-10 in the aqueous humor were tested by microsphere-based flow cytometry. Fluorescent quantitative polymerase chain reaction (PCR) was performed to determine the nucleic acid copy number of cytomegalovirus (CMV), varicella-zoster virus, and herpes simplex virus (HSV).

### 2.5. Statistical analysis

SPSS 22.0 (IBM Inc., Armonk, NY) and GraphPad Prism 9 (GraphPad Inc., San Diego, CA) were used for statistical analyses. Measurement data are expressed as mean ± standard deviation (mean ± SD). Fisher’s exact test was used for categorical variables. Continuous variables such as age, disease duration, IOP, and corneal endothelial cell density were compared between the groups using the Student’s *t*-test. Spearman’s analysis was used for correlation analysis between factors. Since the cytokine concentrations did not conform to a normal distribution, Mann-Whitney U test was used to compare the differences between the two groups. Binary logistic regression analysis was used for cytokines owing to multicollinearity between cytokines. Differences with *p* < 0.05 were considered statistically significant.

## 3. Results

### 3.1. Patient characteristics

The clinical characteristics of the patients are shown in Table 1. There were 8 males (88.9%) and 1 female (11.1%) in PSS group, with 6 right eyes (66.67%) and 3 left eyes (33.33%). There were 6 males (85.7%) and 1 female (14.3%) in OAG secondary to PSS group, with 4 right eyes (57.14%) and 3 left eyes (42.86%). There was no significant difference in age, sex, disease duration, or eye laterality between the two groups (*p* > 0.05). There were no significant differences in ocular characteristics and performance, including peak IOP, CEC density, baseline visual acuity, cup-to-disk ratio, RNFL thickness, visual field defects, keratic precipitates, or aqueous flares between the two groups (*p_1_* = 0.5638, *p_2_* = 0.3059, *p_3_* = 0.7166, *p_4_* = 0.3328, *p_5_* = 0.9868, *p_6_* = 0.5500, *p*_7_ = 0.5962, *p_8_* = 0.5846, respectively).

**TABLE 1:** Demographic and clinical data of all patients.

	PSS (*n* = 9)	PSS + OAG (*n* = 7)	*p*-value^a^
Age, years (mean ± SD)	46.56 ± 14.37	41.86 ± 10.79	0.4839
Sex (%)			1.0000
Male	8 (88.90)	6 (85.70)	-
Female	1 (11.10)	1 (14.30)	-
Disease duration, years (mean ± SD)	4.72 ± 4.39	3.82 ± 7.18	0.7603
Affected eye (%)			1.0000
Right eye	6 (66.67)	4 (57.14)	-
Left eye	3 (33.33)	3 (42.86)	-
CMV (+)	5 (55.60)	7 (100.00)	0.0885
Peak IOP, mmHg (mean ± SD)	39.89 ± 14.12	43.57 ± 9.50	0.5638
CEC density,/mm^2^ (mean ± SD)	2043.11 ± 774.69	2375.57 ± 314.52	0.3059
Baseline vision (mean ± SD)	0.54 ± 0.27	0.49 ± 0.21	0.7166
Cup-to-disk ratio (mean ± SD)	0.62 ± 0.16	0.71 ± 0.20	0.3328
RNFL thickness, μm (mean ± SD)	82.33 ± 20.56	82.14 ± 24.56	0.9868
Visual field defect	8 (88.89)	5 (71.43)	0.5500
KPs (+)	7 (77.78)	4 (57.14)	0.5962
Tyn (+)	3 (33.33)	1 (14.30)	0.5846

Data are presented as mean ± SD or number of patients (%).

*^a^*Fisher’s exact test was used to compare proportions between PSS and PSS + OAG; Unpaired *t*-test was used to compare continuous variables between PSS and PSS + OAG. PSS, Posner-Schlossman Syndrome; PSS + OAG, patients with PSS and open angle glaucoma; CMV, cytomegalovirus; IOP, intraocular pressure; CEC, corneal endothelial cell; RNFL, retinal nerve fiber layer; KPs, keratic precipitates; Tyn, Tyndall effect.

### 3.2. Statistical analysis of aqueous virus and cytokines

The results of virus nucleic acid testing indicated CMV infection in 5 eyes (55.56%) and HSV infection in 2 eyes (data not shown) among the patients with PSS and CMV infection in all patients with OAG secondary to PSS (100%), with no significant difference in the CMV infection rate between the two groups (*p* = 0.0885). Comparison of CMV-positive and CMV-negative PSS patients (Table 2) showed that the corneal endothelial cell density of CMV-positive PSS patients (1,528.00 ± 546.61/mm^2^) was significantly lower than that of CMV-negative PSS patients (2,687.00 ± 454.95/mm^2^), the difference was statistically significant (*p* = 0.0116). However, there were no significant differences in peak IOP, baseline visual acuity, cup-to-disk ratio, RNFL thickness, visual field defects, keratic precipitates, and aqueous flares between the two groups (*p*_1_ = 0.9810, *p*_2_ = 0.5809, *p*_3_ = 0.2637, *p*_4_ = 0.5641, *p*_5_ = 0.4444, *p*_6_ = 0.1667, *p*_7_ = 1.0000, respectively).

**TABLE 2:** Demographic and clinical data of patients with PSS.

	CMV positive (*n* = 5)	CMV negative (*n* = 4)	*p*-value^a^
Peak IOP, mmHg (mean ± SD)	40.00 ± 14.65	39.75 ± 15.69	0.9810
CEC density,/mm^2^ (mean ± SD)	1528.00 ± 546.61	2687.00 ± 454.95	0.0116*
Baseline vision (mean ± SD)	0.49 ± 0.35	0.60 ± 0.16	0.5809
Cup-to-disk ratio (mean ± SD)	0.68 ± 0.13	0.55 ± 0.19	0.2637
RNFL thickness, μm (mean ± SD)	86.20 ± 23.86	77.50 ± 17.67	0.5641
Visual field defect	5 (100.00)	3 (75.00)	0.4444
KPs (+)	5 (100.00)	2 (50.00)	0.1667
Tyn (+)	2 (40.00)	1 (25.00)	1.0000

Data are presented as mean ± SD or number of patients (%). *^a^*Fisher’s exact test was used to compare proportions between the cytomegalovirus (CMV)-positive and CMV-negative groups; Unpaired *t*-test was used to compare continuous variables between the CMV-positive and CMV-negative groups. PSS, Posner-Schlossman syndrome *p < 0.05.

**FIGURE 1:**
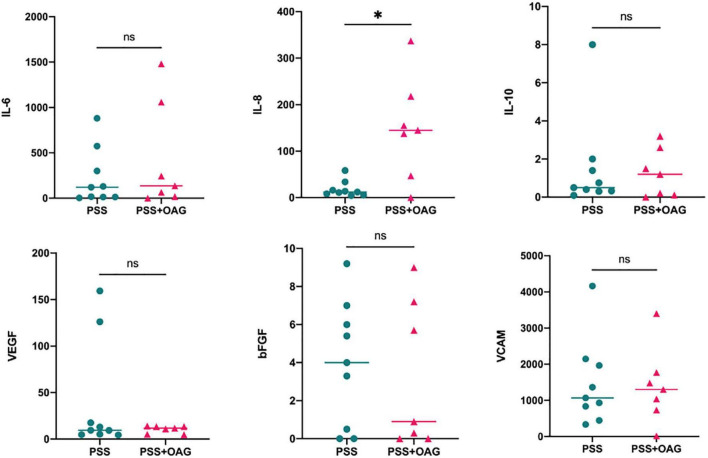
Distribution of cytokine concentrations for PSS and PSS + OAG. The PSS + OAG group had significantly higher cytokine IL-8 concentration levels (145.10 [0.00-337.20]pg/ml) than the PSS group (12.20 [4.70-58.50]pg/ml). The concentration levels of IL-6, IL-10, VEGF, bFGF, and VCAM did not differ significantly between the groups. *p<0.05. bFGF, basic fibroblast growth factor; IL, interleukin; OAG, open angle glaucoma; PSS, Posner-Schlossman syndrome; VCAM, vascular cell adhesion molecule; VEGF, vascular endothelial growth factor.

The results of aqueous humor cytokine assays at the first occurrence of PSS in the two groups are shown in Table 3. Among them, the median concentration of bFGF (4.00 pg/ml) in the PSS group was higher than the standard reference value, the median concentration of IL-8 (145.10 pg/ml) in patients with OAG secondary to PSS was higher than the standard reference value. The median concentrations of IL-6 (121.70 pg/ml, 135.80 pg/ml) and VCAM (1071.00 pg/ml, 1304.00 pg/ml) in the PSS and OAG secondary to PSS groups were higher than the standard reference values. The non-parametric rank-sum test (Mann-Whitney U test) was used to compare the aqueous humor cytokine concentrations between the two groups. The level of IL-8 concentration in the aqueous humor of patients with OAG secondary to PSS (145.10 [0.00-337.20] pg/ml) was significantly higher than that in the PSS group (12.20 [4.70-58.50] pg/ml); the difference was statistically significant (*p* = 0.0229). There were no significant differences in IL-6 (*p* = 0.4914), IL-10 (*p* = 0.9578), VEGF (*p* = 0.9578), bFGF (*p* = 0.7898), and VCAM (*p* = 0.9678) between the two groups (Figure 1).

**TABLE 3:** Concentrations of aqueous cytokine levels.

Cytokines pg/ml	PSS (*n* = 9)	PSS + OAG (*n* = 7)	Standard reference value^b^	PSS VS PSS + OAG *p*-value^a^
	**Median (range)**	**Median (range)**		
IL-6	121.70 (3.30-881.40)^↑^	135.80 (0.00-1480.80)^↑^	1.00-50.00	0.4914
IL-8	12.20 (4.70-58.50)	145.10 (0.00-337.20)^↑^	0.00-20.00	0.0229*
IL-10	0.50 (0.09-8.00)	1.20 (0.00-3.20)	0.00-5.00	0.9578
VEGF	9.50 (4.47-159.40)	11.78 (4.83-14.10)	0.00-40.00	0.9578
bFGF	4.00 (0.0-9.2)^↑^	0.90 (0.00-9.00)	0.00-1.00	0.7898
VCAM	1,071.00 (334.30-4,165.10)^↑^	1304.00 (20.40-3405.80)^↑^	200.00-1000.00	0.9678

Data are presented as median (range) (pg/ml).

*^a^*Mann-Whitney U test was used to compare cytokine concentrations between PSS and PSS + OAG.

*^b^*Standard reference value is the normal healthy human aqueous cytokine concentration given by the detection mechanism.

IL-6, interleukin-6; IL-8, interleukin-8; IL-10, interleukin-10; VEGF, vascular endothelial growth factor; bFGF, basic fibroblast growth factor; VCAM, vascular cell adhesion molecule.

^↑^The median cytokine concentration was above standard reference value.

*p < 0.05.

### 3.3. Correlations between aqueous cytokines

**FIGURE 2:**
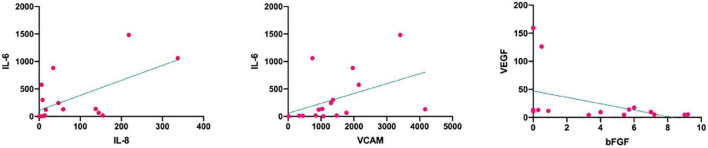
Correlations between the cytokines in the two groups. IL-8 and IL-6 (ρ = 0.5412, *p* = 0.0304) and IL-6 and VCAM (ρ = 0.5853, *p* = 0.0172) were significantly positively correlated. bFGF and VEGF (ρ = –0.4978, *p* = 0.0497) were significantly negatively correlated. bFGF, basic fibroblast growth factor; IL, interleukin; VCAM, vascular cell adhesion molecule; VEGF, vascular endothelial growth factor.

To investigate the correlation between cytokines in aqueous humor, Spearman’s correlation analysis was performed on the concentration levels of six cytokines (Table 4). There was a significant positive correlation between IL-6 and IL-8 (ρ = 0.5412, *p* = 0.0304), IL-6 and VCAM (ρ = 0.5853, *p* = 0.0172); There was a significant negative correlation between bFGF and VEGF (ρ = −0.4978, *p* = 0.0497) (Figure 2). IL-10 was not correlated with other cytokines.

**TABLE 4:** Correlations between aqueous cytokines.

ρ /*p* value^a^	IL-6	IL-8	IL-10	VEGF	bFGF	VCAM
IL-6		0.5412*	0.1971	0.1000	-0.0696	0.5853*
IL-8	0.0304^a^		0.4735	0.2147	0.0578	0.3529
IL-10	0.4645	0.0639		0.0029	0.3467	0.3324
VEGF	0.7125	0.4246	0.9914		-0.4978*	0.0088
bFGF	0.7978	0.8317	0.1884	0.0497^c^		-0.1126
VCAM	0.0172^b^	0.1800	0.2085	0.9741	0.6780	

^a^Spearman’s correlation test was used to calculate the correlation coefficient ρ and p values for cytokines in the two groups. The *p* values are shown on the lower left side and the correlation coefficients ρ on the upper right side of the table.

*p*^a^ < 0.05, IL-8 vs. IL-6; *p*^b^ < 0.05, IL-6 vs. VCAM; *p*^c^ < 0.05, bFGF vs. VEGF.

ρ values with significance < 0.05 are marked with *.

bFGF, basic fibroblast growth factor; IL, interleukin; VCAM, vascular cell adhesion molecule; VEGF, vascular endothelial growth factor.

### 3.4. Association of PSS concomitant OAG with aqueous cytokines

**FIGURE 3:**
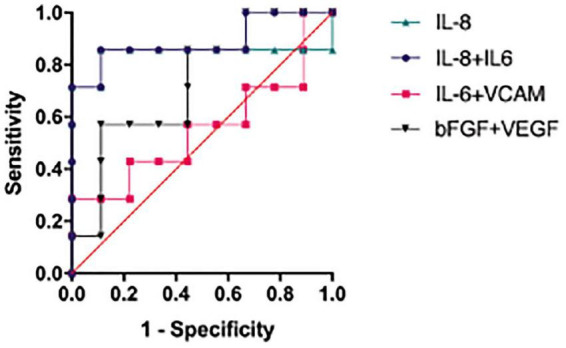
Accuracy of correlated cytokines in predicting OAG secondary to PSS. IL-8 together with IL-6 (*p* = 0.0095) had higher accuracy in predicting OAG secondary to PSS than IL-8 alone (*p* = 0.0229). IL-6 with VCAM (*p* = 0.7110) and bFGF with VEGF (*p* = 0.1248) had no predictive value. bFGF, basic fibroblast growth factor; IL, interleukin; OAG, open angle glaucoma; PSS, Posner-Schlossman syndrome; VCAM, vascular cell adhesion molecule; VEGF, vascular endothelial growth factor.

Finally, we analyzed the possibility of these aqueous humor cytokine concentration levels for predicting OAG secondary to PSS. Binary logistic regression analysis was performed for IL-8, which had a significant difference, and between IL-8 and IL-6, IL-6 and VCAM, bFGF and VEGF, which were significantly correlated (Table 5). Of the four predictors, increased IL-8 concentration and the simultaneous increase of IL-8 and IL-6 concentration levels were significantly correlated with OAG secondary to PSS. The simultaneous increase of IL-8 and IL-6 concentrations (*p* = 0.0095) was more accurate than that of IL-8 alone (*p* = 0.0229) (Figure 3). This indicates that the simultaneous increase of IL-8 and IL-6 concentrations may be a better predictor of OAG secondary to PSS. IL-6 and VCAM (*p* = 0.7110), bFGF and VEGF (*p* = 0.1248) were not significantly correlated with OAG secondary to PSS.

**TABLE 5:** Predictive accuracy of aqueous cytokines.

Predictor	AUC	Std. Error	*p*-value^a^	95% Cl
IL-8	0.8413	0.1322	0.0229*	0.5822-1.000
IL-8 & IL-6	0.8889	0.0952	0.0095*	0.7024-1.000
IL6 & VCAM	0.5556	0.1567	0.7110	0.2484-0.8627
bFGF & VEGF	0.7302	0.1299	0.1248	0.4755-0.9848

^a^Binary logistic regression analysis was used to detect the accuracy of relevant cytokines for predicting open angle glaucoma secondary to Posner-Schlossman Syndrome.

AUC, area under curve; Std. error, standard error; Cl, confidence interval.

*p < 0.05..

## 4. Discussion

Posner-Schlossman syndrome is a disease characterized by recurrent episodes of non-granulomatous anterior uveitis and elevated IOP, and its causes include CMV infection, immune factors, autonomic dysfunction, and vascular endothelial dysfunction (9, 16, 17). However, its exact causes remain inconclusive. PSS can lead to secondary chronic glaucoma, and its duration may be one of the risk factors for glaucoma progression, but the causes of OAG secondary to PSS remain poorly understood. Previous studies have found differences in clinical features and aqueous humor cytokines between patients with PSS and OAG. Nevertheless, no studies have been performed to analyze the risk factors in patients with OAG secondary to PSS by aqueous humor testing.

First, we retrospectively analyzed the clinical characteristics of patients with PSS and patients with OAG secondary to PSS, the aqueous humor virus nucleic acid, and aqueous humor cytokines at the time of the first occurrence of PSS in the two groups. Comparison of clinical data revealed no significant differences in age, sex, disease duration, and eye laterality between the groups. There were no significant differences in peak IOP, corneal endothelial cell density, baseline visual acuity, cup-to-disk ratio, RNFL thickness, visual field defects, keratic precipitates, and anterior uveitis between patients with PSS and OAG secondary to PSS. In addition, there was also no significant difference in the clinical characteristics of the two groups of patients at the first occurrence of PSS (data not shown). Liu et al. found that more obvious decreased vision and visual field defects in patients with glaucoma secondary to uveitis (18), but we did not find a significant difference between the two groups for these parameters. This inconsistency may be attributable to the temporary elevation of IOP and transient loss of visual acuity during the active phase in PSS patients, whereas patients with OAG secondary to PSS have not yet developed severe visual impairment. In addition, Moore et al. found a higher-than-expected increase in RNFL thickness in patients with glaucoma secondary to uveitis (19), and Gangaputra et al. found a relatively high rate of inconsistency in cup-to-disk ratio assessment by clinicians (20); hence, the RNFL thickness and cup-to-disk ratio may not truly reflect the severity of glaucoma secondary to uveitis.

In this study, we found that CMV infection rate was 55.56% in patients with PSS and 100% in patients with OAG secondary to PSS. CMV, a member of the human herpesvirus family, has been proven to be a pathogen causing anterior uveitis and cornea endodermatitis. Studies have shown a high prevalence of ocular CMV infection in patients with PSS (13, 21–23). Chee et al. showed that up to 52.2% of patients with PSS were CMV-positive (21), which is generally consistent with our findings. Comparison of CMV-positive and CMV-negative PSS patients showed that the former had significant loss of corneal endothelial cells and the density was significantly lower than the latter, which may be associated with CMV attacking the trabecular meshwork endothelial cells and corneal endothelial cells, resulting in increased IOP and corneal endothelial cell loss (24, 25). Su et al. found that ganciclovir inhibits CMV viral activity and thus reduces the damage of trabecular meshwork endothelium and corneal endothelium (26), thereby decreasing the risk of advanced glaucoma. In this study, there was no significant difference in the CMV infection rate between the two groups, which may be related to the limited number of patients enrolled and the relatively short follow-up period, However, in clinical diagnosis and treatment, we generally found that the CMV-positive PSS patients may have higher IOP, poorer baseline visual acuity, larger cup-to-plate ratio, and more severe ocular inflammation, which was basically consistent with the trend of previous studies (26, 27). Therefore, the risk of OAG in CMV-positive PSS patients cannot be excluded.

The Interleukin family includes important inflammatory cytokines, such as IL-1, IL-6, IL-8, IL-10, and IL-35 (28). IL-8 is a pro-inflammatory factor with immune and vascular functions that induces inflammation and promotes angiogenesis by inducing neutrophil chemotaxis and stimulating phagocytosis (29–32). IL-8 has been shown to play an important role in several types of uveitis. An investigation by Kuchtey et al. showed that IL-8 concentration in the aqueous humor was significantly higher in patients with OAG and it is closely related to the severity of the disease (33), suggesting that glaucoma may be associated with immune activation. Intraocular IL-8 is secreted by lens epithelial cells and trabecular meshwork endothelial cells, and increased IL-8 concentration is positively correlated with IOP, indicating that IL-8 is involved in trabecular meshwork regulation of the aqueous outflow pathway (34). The significantly increased IL-8 concentration in patients with OAG secondary to PSS in the present study suggests that immune activation mediates chronic oxidative stress and ischemic injury to trabecular meshwork endothelial cells, ultimately leading to trabecular meshwork loss and dysfunction. IL-6 is a multifunctional pro-inflammatory factor produced by T cells, monocytes, and macrophages. It is the most physiologically potent inflammatory cytokine known, with markedly increased in idiopathic uveitis and ocular infections (35, 36). Engel et al. found that IL-6 concentration increased significantly in OAG patients with poor IOP control after trabeculectomy (37), showing that IL-6 may also be a negative factor in the development of OAG. IL-8 and IL-6 have been shown to be regulated by the NF-kB pathway and synergistically play a key role in immune response (38). In this study, IL-8 concentration in patients with OAG secondary to PSS was higher than the standard reference value and significantly higher than in the PSS group. In addition, the simultaneous increase of IL-8 and IL-6 concentrations were significantly associated with OAG secondary to PSS. Notably, simultaneous increase of IL-8 and IL-6 concentrations had a stronger correlation with OAG development than the increase of IL-8 alone. Thus, we propose that simultaneous increase of IL-8 and IL-6 further enhances the inflammatory response, exacerbates inflammatory damage of the trabecular meshwork, and accelerates the development of OAG through related immune mechanisms. Therefore, the simultaneous increase of IL-8 and IL-6 concentration levels is more valuable for predicting OAG secondary to PSS than that of IL-8 alone. It can be used as a risk index for predicting OAG secondary to PSS, which may be of great implications for early diagnosis and intervention.

Vascular cell adhesion molecule is a cell adhesion molecule associated with the stability of the blood-eye barrier. Under normal physiological conditions, vascular endothelial cells secrete less VCAM, and the increased level of VCAM indicates inflammation (39). In this study, IL-6 and VCAM were increased in both groups compared to the standard reference values, suggesting a more severe inflammatory reaction and intraocular tissue edema. VEGF and bFGF are both heparin-binding platelet-derived growth factors that induce angiogenesis *in vivo* and vitro, and they synergistically promote endothelial cell survival and proliferation (40). We observed a negative correlation between VEGF and bFGF concentrations without synergism, indicating that the overall neovascularization tendency was not obvious in either patient with PSS or with OAG secondary to PSS.

In conclusion, the simultaneous increase of IL-8 and IL-6 concentrations may be a high risk factor to accelerate OAG secondary to PSS, with IL-8 playing a more important role. Simultaneous increase of IL-8 and IL-6 can be used as a more reliable cytokine marker for predicting OAG secondary to PSS. However, the high possibility of OAG in CMV-positive PSS patients in the future should not be ignored, and regulation of IL-8 and IL-6 levels may be a new intervention to prevent OAG secondary to PSS. Certainly, there are some limitations in this study: it was a retrospective analysis, the sample size of this study was small, and long-term follow-up of patients was not performed. In addition, limited by the safety concerns of anterior paracentesis and the high cost of aqueous humor testing, large-sample randomized controlled trials are needed to further confirm these results in the further, so as to explore the potential risk factors for OAG secondary to PSS and implement early risk control, intervention, and treatment.

## Data availability statement

The original contributions presented in this study are included in the article/supplementary material, further inquiries can be directed to the corresponding authors.

## Ethics statement

The studies involving human participants were reviewed and approved by Institutional Review Board of the Eye Hospital of Nanjing Medical University. The patients/participants provided their written informed consent to participate in this study.

## Author contributions

JL and KL carried out the studies and drafted the manuscript. JL and YJ participated in data collection. KL and JY participated in the study design and guidance. All authors read and approved the final manuscript.
